# Sorafenib modulates the radio sensitivity of hepatocellular carcinoma cells *in vitro* in a schedule-dependent manner

**DOI:** 10.1186/1471-2407-12-485

**Published:** 2012-10-22

**Authors:** Qiaoqiao Li, Yonghong Hu, Mian Xi, Liru He, Lei Zhao, Mengzhong Liu

**Affiliations:** 1 Department of Radiation Oncology, SunYat-sen University Cancer Center Guangzhou, 651 Dongfeng Road East, Guangzhou, 510060, China; 2 State Key Laboratory of Oncology in South China, Guangzhou, China

**Keywords:** Hepatocellular carcinoma, Radiation, Sorafenib, Apoptosis, DNA damage repair

## Abstract

**Background:**

Hepatocellular carcinoma (HCC) has a high incidence and mortality. Radiotherapy and sorafenib have proven effective for HCC. Here, we investigated whether sorafenib modulated the response of HCC cells to irradiation *in vitro,* effect of timing of sorafenib, and the underlying mechanisms.

**Methods:**

Cell viability of the HCC cell lines, SMMC-7721 and Bel-7402, was examined by the 3-(4,5-dimethylthiazol-2-yl)-5(3-carboxymethoxyphenyl)-2(4-sulfophenyl)-2 H-terazolium (MTT) assays. Clonogenic growth assays of SMMC-7721 and Bel-7402 were determined by colony formation assays. DNA damage was assessed by monitoring γ-HAX foci in irradiated cells with immunofluorescence microscopy, and cell cycle distribution changes were examined by flow cytometry. Effects of sorafenib (15 μM) added 30 min prior to radiation (pre-irradiation sorafenib) of SMMC-7721 and BEL-7402 or 24 h post-irradiation (post-irradiation sorafenib) on irradiated SMMC-7721 and BEL-7402 cells were compared to those of radiation alone or no treatment.

**Results:**

The effect of sorafenib was dependent on its time of addition in relationship to irradiation of cells. Pre-irradiation sorafenib did not significantly affect the viability of SMMC-7221 and BEL-7402 cells compared with irradiation treatment alone. In contrast, post-irradiation sorafenib increased the sensitivity of irradiated SMMC-7221 and BEL-7402 cells significantly in a time-dependent manner. Pre-irradiation sorafenib significantly increased the surviving fraction of SMMC-7221 and BEL-7402 cells in clonogenic assays whereas post-irradiation sorafenib significantly reduced the surviving fractions of SMMC-7221 and BEL-7402 cells. SMMC-7721 cells treated with sorafenib 30 min before irradiation had significantly fewer cells with γ-H2AX foci (23.8 ± 2.9%) than SMMC-7721 cells receiving radiation alone (59.9 ± 2.4; *P* < 0.001). Similarly, BEL-7402 cells receiving sorafenib prior to irradiation had significantly fewer cells with γ-H2AX foci (46.4 ± 3.8%) than those receiving radiation alone (25.0 ± 3.0%; P < 0.001). In addition, irradiation (6 Gy) caused a significant increase in the percentage of both SMMC-7721 and BEL-7402 cells in G2/M at 12 to 16 h post irradiation, which was markedly delayed by pre-irradiation sorafenib.

**Conclusions:**

Sorafenib combined with irradiation exerted a schedule-dependent effect in HCC cells *in vitro*, which has significant implications for the combined use of sorafenib and radiotherapy for HCC patients.

## Background

Primary hepatocellular carcinoma is the 6^th^ most common malignancy in the world and ranks 3^rd^ among causes of cancer-related death. Hepatocellular carcinoma is prevalent in China and accounts for 55% of all hepatocellular carcinoma cases in the world [1]. Despite the best therapeutic regimen currently available, hepatocellular carcinoma has a dismal outcome with the five-year survival rate of 3% -10% for metastasized HCC and 28% for locally confined HCC. Approximately 80% of hepatocellular carcinoma patients have inoperable cancer at the time of diagnosis [2]. The median survival for patients with inoperable hepatocellular carcinoma is generally about 6 months [2].

Recently, adjuvant radiotherapy has shown promise as a treatment for inoperable hepatocellular carcinoma with a response rate of 30 ~ 67% [3-5]. Since radiotherapy is limited by poor tolerance of radiation in adjacent normal tissues, and regional radiotherapy has no tangible effect on intrahepatic and distant metastasis, agents that boost the sensitivity to radiotherapy are sought. Sorafenib is a multikinase inhibitor with anti-proliferative and anti-angiogenic effects. It inhibits the activity of the serine/threonine kinases c-Raf and B-Raf; the mitogen-activated protein kinases MEK and ERK; vascular endothelial growth factor receptors (VEGF); platelet-derived growth factor receptors (PDGFR); the cytokine receptor c-KIT; the receptor tyrosine kinases Flt-3 and RET; and the Janus kinase/signal transducer and activator of transcription (JAK/STAT) pathway [6]. Phase III clinical studies have shown that sorafenib is efficacious in patients with advanced hepatocellular carcinoma [7,8], and sorafenib is the most recent drug approved for hepatocellular carcinoma. However, sorafenib only modestly improves the outcome of hepatocellular carcinoma patients, prolonging the median survival of patients with inoperable hepatocellular carcinoma by less than 3 months [7]. Mechanistically, sorafenib increases apoptosis of the hepatocellular carcinoma cells, PLC/PRF/5 and HepG2 cells [9] as well as some breast cancers, colorectal carcinomas, osteosarcomas, and glioblastomasbut not all types of tumor cells [10]. Sorafenib may augment radiotherapy of HCC because administration of sorafenib post-irradiation markedly potentiated the inhibitory effect of irradiation on growth of mouse colorectal cancer xenografts compared to irradiation alone [10]. However, the combination of irradiation and concurrent sorafenib administration had no significant effect on tumor growth [10]. Suen et al. [11] investigated the combined effect of sorafenib and irradiation on colorectal cancer cells: only sorafenib given post irradiation augments the inhibitory effects of irradiation on clonogenic growth. Interestingly, three renal cell carcinoma patients who relapsed under sorafenib were subsequently co-administered radiotherapy [12]. Sorafenib treatment was administered both prior to and concurrently with radiation [12]. In these three RCC cases, the tumor mass shrunk, pain diminished or was abolished, and patients reported no late side effects [12].

We hypothesized that sorafenib may also boost the efficacy of irradiation on HCC in a schedule-dependent manner. A case report of a patient with inoperable HCC who was initially treated with sorafenib provides support of interaction between radiotherapy and sorafenib during treatment of HCC [13]. The patient’s history included sorafenib treatment, its subsequent discontinuation due to side effects, unchecked tumor growth, treatment with both radiotherapy and sorafenib, tumor shrinkage, and the recurrence of sorafenib-related rash [13]. Currently, optimization of combined irradiation and sorafenib in hepatocellular carcinoma has not been described, and the mechanisms of irradiation enhanced by sorafenib are still ambiguous. We investigated the effect of combined radiotherapy and sorafenib on two hepatocellular carcinoma cell lines, SMMC-7721 and BEL-7402, and the underlying mechanisms of interaction.

## Methods

### Cell lines and agents

Human hepatocellular carcinoma cell lines, SMMC-7721 and Bel-7402, were obtained from Nanfang Hospital of Southern Medical University, Guangzhou, Guangdong, China [14], and were cultured in RPMI-1640 supplemented with 10% heat-inactivated fetal bovine serum (FBS) (Hyclone, Logan City, Utah) at 37°C in a humidified atmosphere containing 5% CO_2_. Sorafenib (Bayer, Leverkusen, Germany) was dissolved in dimethyl sulfoxide (DMSO) to a stock concentration of 25 mmol/L and stored at -20°C.

### The 3-(4,5-dimethylthiazol-2-yl)-5(3-carboxymethoxyphenyl)-2(4-sulfophenyl)-2 H-terazolium (MTT) assays

The MTT (3-(4,5-dimethylthiazol-2-yl)-5(3-carboxymethoxyphenyl)-2(4-sulfophenyl)-2 H-terazolium) assays (Promega, Madison, WI) were performed as instructed by the manufacturer to assess cell viability. Briefly, SMMC-7721 (3 Χ 10^3^ cells/well) and BEL-7402 cells (4 ×10^3^) were seeded into 96-well plates in quadruplicate. After incubation for 1 d, cells were treated with sorafenib 30 min before (pre-irradiation sorafenib) or 24 h following irradiation (post-irradiation sorafenib). Cells were irradiated at the indicated doses using a ^60^Co irradiator. Cell viability was measured on d0 to d6 after irradiation. Absorbance values were shown as the percentage of the treated samples relative to the controls which received neither irradiation nor sorafenib. Inhibition of cell growth was measured as the percentage of viable cells relative to the controls, which was calculated as follows: % of viable cells = OD_T_/OD_C_ x 100%, where OD_T_ is the average OD value of the treatment samples, and OD_C_ is the average OD value of the control samples. Results were analyzed using the CalcuSyn software program (Biosoft, Cambridge, UK). Combination indices (CI) were used to assess the interaction between the two treatment modalities.

### Apoptotic study and cell cycle analysis

SMMC-7721 and BEL-7402 cells were irradiated, treated with sorafenib for 30 min followed by irradiation (pre-irradiation sorafenib), or irradiated and treated 24 h later with sorafenib (post-irradiation sorafenib). Apoptosis was detected in cells washed with phosphate buffered saline (PBS) at 48 h post-irradiation (irradiated controls, pre-irradiation sorafenib) or 72 h post-irradiation (post-irradiation sorafenib) by staining with annexin V and propidium iodide as instructed by the manufacturer (BD Biosciences, Franklin Lake, NJ). Stained cells were analyzed by flow cytometry with a FACSCalibur flow cytometer (BD Biosciences). For cell cycle analysis, treated cells were washed once with PBS, trypsinized, washed in PBS with 2% FBS, fixed in ice-cold ethanol for at least 1 h, washed, stained with propidium iodide (30 μg/mL), and treated with RNase (0.6 mg/ml) in PBS plus 0.5% (v/v) Tween 20 and 2% FBS. Stained cells were analyzed on a FACSCalibur flow cytometer (BD Biosciences) by using the CellQuest software. Mod-Fit program (Verity Software House Inc., Topsham, ME) was used to analyze the cell-cycle profiles.

### Colony formation assays

This procedure was performed as previously described [15]. Briefly, cells were irradiated at a dose of 0, 2, 4, and 8 Gy alone or in combination with sorafenib administered 30 min prior to (pre-irradiation sorafenib) or 24 h following irradiation (post-irradiation sorafenib). After incubation of 12 d (SMMC-7721) or 14 d (BEL-7402), cells were stained with 0.5% crystal violet in absolute ethanol, and colonies containing more than 50 cells were counted under a dissection microscope. Clonogenic survival curves were constructed by fitting the average survival levels. Subsequent experiments utilized a radiation dose of 6 Gy because the percentage of cells remaining after 8 Gy (SMMC-7721: 0.9-4%; BEL-7402: 2-5%) was too low for analysis. SMMC-7721 and BEL-7402 cells in subsequent experiments received one of the four treatments: (a) none (control), (b) 6 Gy radiation, (c) 15 μM sorafenib 30 min before 6 Gy radiation, or (d) 6 Gy radiation followed 24 h later with 15 μM sorafenib.

### DNA damage immunofluorescence microscopy

Immunofluorescence microscopy was done as previously described [16]. Rabbit anti-γ-H2AX antibody (serine 139; Abcam, Cambridge, MA), and secondary antibodies Alex Fluor 488 goat anti-rabbit IgG (Invitrogen, Carlsbad, CA) were used. Nuclear staining was done by using 4’, 6-diamidino-2-phenylindole (DAPI) (Vector Laboratories, USA). A cell containing more than 10 γ-H2AX foci was considered to be positive for damages to DNA.

### Cell cycle G2/M distribution assay

After the indicated time period, cells were rinsed with PBS, fixed with 70% ethanol, and incubated overnight at -20°C. Fixed cells were washed and suspended in 500 μl of staining solution (50mcg/ml of propidium iodide, 100mcg/ml RNAase and 0.2% Triton X-100) for 30 min. The fluorescence associated with PI-bound DNA was measured by flow cytometry (Beckman Coulter, cytomics FC 500, CA). Cell cycle profiles of G2/M phase were calculated using MultiCycle software.

### Cell proliferation assays

SMMC-7721 and BEL-7402 cells were plated at 1 x 10^3^ cells per well in collagen-coated 96-well plates. Cell proliferation assays were performed by using the Cell Counting Kit-8 (CCK8) (Dojindo, Kumamoto, Japan) according to the manufacturer's protocol. Briefly, a 10 μL of CCK-8 solution was added to each well and incubated at 37°C for 2 h in a humidified CO_2_ incubator. Optical density (OD) was measured at 450 nm using a Microplate Reader (Bio-Tek Instruments, Winooski, VT) and the proliferation index was calculated as the experimental OD value/control OD value. Each experiment was done in quadruplicate and at least three times independently.

### Apoptosis assays

After incubation for 0 h, 24 h, or 48 h after sorafenib treatment, cells were harvested, rinsed, and stained with Annexin V-FITC and propidium iodide, as previously described [17].

#### Statistical analyses

Normally distributed continuous variables were compared by one-way analysis of variance (ANOVA). When a significant difference between groups was apparent, multiple comparisons of means were performed using the Dunnett test. Data are presented as mean ± standard deviation (SD). All statistical assessments were two-sided and evaluated at the 0.05 level of significant difference. Statistical analyses were performed using SPSS 15.0 statistics software (SPSS Inc, Chicago, IL).

## Results

### Sorafenib modulated radio sensitivity of hepatocellular carcinoma cells in a schedule-dependent manner

**Figure 1:**
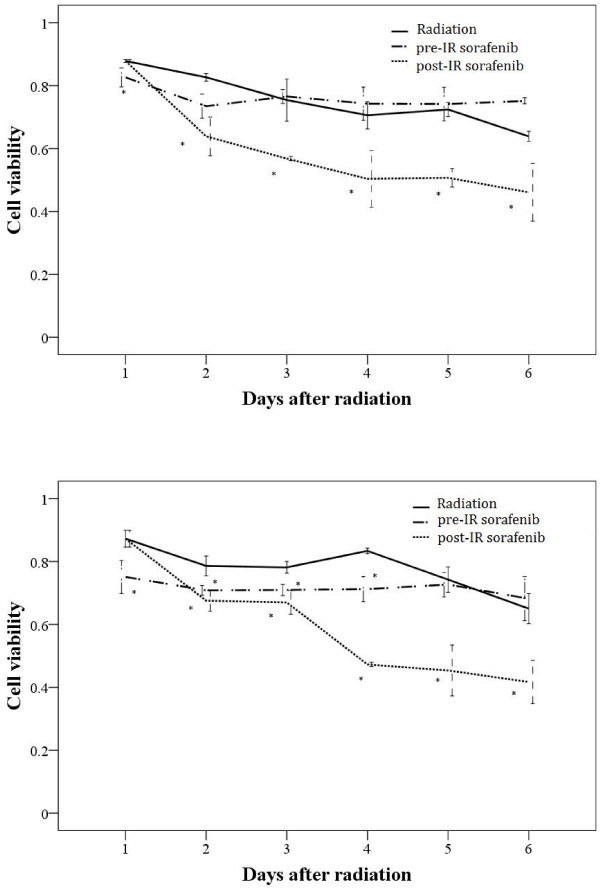
**Effect of sorafenib treatmenton cell viability ofirradiated SMMC-7721 (A) andBEL-7402 cells (B).** Cells were treated with radiation, sorafenib 30 min prior to irradiation (pre-IR sorafenib), or 24 h post irradiation (post-IR sorafenib), and MTT assays were performed to measure the viability of irradiated, treated cells. Cell viability was significantly lower in the post-irradiation sorafenib group versus the irradiation or pre-irradiation sorafenib group. Mean values were compared by using ANOVA. Mean ± SD (n = 3). *P < 0.05 vs. Radiation group.

To investigate whether sorafenib modulated the response of hepatocellular carcinoma cells to radiation, we added sorafenib 30 min prior to or 24 h following irradiation of hepatocellular carcinoma cells SMMC-7721 and BEL-7402 and measured cellular viability by MTT for 6 days (Figure 1). Pre-irradiation sorafenib did not significantly affect the viability of SMMC-7221 and BEL-7402 cells (Figure 1A and 1B) (*P* > 0.05). In contrast, post-irradiation sorafenib reduced the sensitivity of irradiated SMMC-7221 and BEL-7402 cells significantly in a time-dependent manner (Figure 1A and 1B) (*P* < 0.05). These findings suggested that sorafenib modulated the radio sensitivity of hepatocellular carcinoma cells in a schedule-dependent manner *in vitro*.

**Figure 2:**
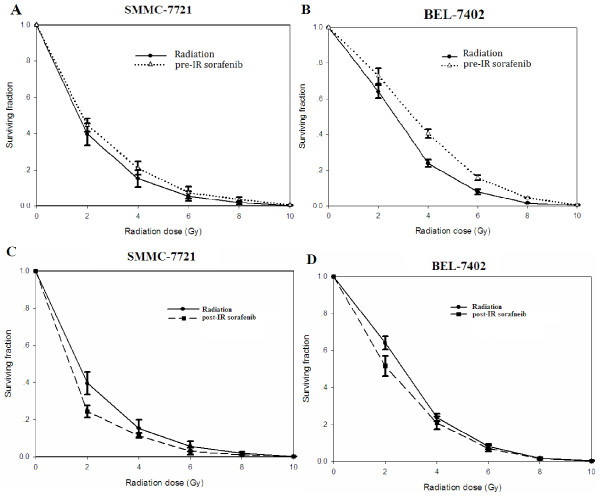
**Clonogenic survival of humanhepatocellular carcinoma cells SMMC-7721(A, C) and BEL-7402(B, D) after irradiationwith or without sorafenib.A**, **B** Sorafenib (15 mM) was added 30 min prior to irradiation of cells (pre-IR sorafenib). **C**, **D**. Cells were irradiated (0-10 Gy) and sorafenib was added 24 h post irradiation (post-IR sorafenib). Survival fraction (SF) was calculated by using the mean plating efficiency (PE) of untreated cells as the denominator to illustrate independent cytotoxic effects of sorafenib; linear quadratic (LQ) equation was fitted to data.

To further assess the effect of sorafenib on the radio sensitivity of HCC cell lines, we performed clonogenic assays. Radiation caused a dose-dependent cytotoxic effect on SMMC-7221 and BEL-7402 cells with less than 20% of cells surviving at 4 Gy and less than 0.1% of cells surviving at 10 Gy. The surviving fraction of SMMC-7221 and BEL-7402 cells was 0.15 ± 0.05 and 0.24 ± 0.02, respectively, at an irradiation dose of 4 Gy. Pre-irradiation sorafenib significantly increased the surviving fraction of SMMC-7221 and BEL-7402 cells: for example, sorafenib increased survival of irradiated (4 Gy) SMMC-7221 to 0.21 ± 0.04 and irradiated (4 Gy) BEL-072 to 0.40 ± 0.03 (Figure 2A and 2B; Table 1) (*P* < 0.05 in both). These data suggested that sorafenib given prior to irradiation rendered hepatocellular carcinoma cells more radio resistant. By contrast, post-irradiation sorafenib added 24 hr post irradiation (4 Gy) decreased the surviving fraction of SMMC-7221 to 0.11 ± 0.01, and that of BEL-7402 cells to 0.21 ± 0.03 (Figure 2C and 2D, respectively; Table 1) (*P* < 0.05 for both). These data indicated that sorafenib given 24 h post irradiation increased the radio sensitivity of hepatocellular carcinoma cells. The above findings altogether suggested that sorafenib exerted a schedule-dependent effect on the sensitivity of hepatocellular carcinoma cells to radiation.

**Table 1:** **Mean values for and(and standard errors ofthe means) calculated byfitting the LQ equationto clonogenic survival**

	**α**	**SEM**	**β**	**SEM**
**SMMC-7721**				
IR	-1.271	0.028	0.866	0.020
IR ± Sorafenib pre	-1.295	0.021	0.850	0.011
(Sorafenib delivered 30 min pre-IR)				
IR ± Sorafenib post	-1.145	0.035	0.927	0.017
(Sorafenib delivered 24 h post-IR)				
**BEL-7402**				
IR	-1.384	0.013	0.804	0.007
IR ± Sorafenib pre	-1.412	0.014	0.785	0.008
(Sorafenib delivered 30 min pre-IR)				
IR ± Sorafenib post	-1.331	0.026	0.831	0.136
(Sorafenib delivered 24 h post-IR)				

Abbreviations: *LQ*: linear quadratic; *IR*: irradiation; *SEM*: standard error of the mean.

### Pre-radiation sorafenib increased ability of irradiated hepatocellular carcinoma cells to subsequently repair DNA damage *in vitro*

**Figure 3:**
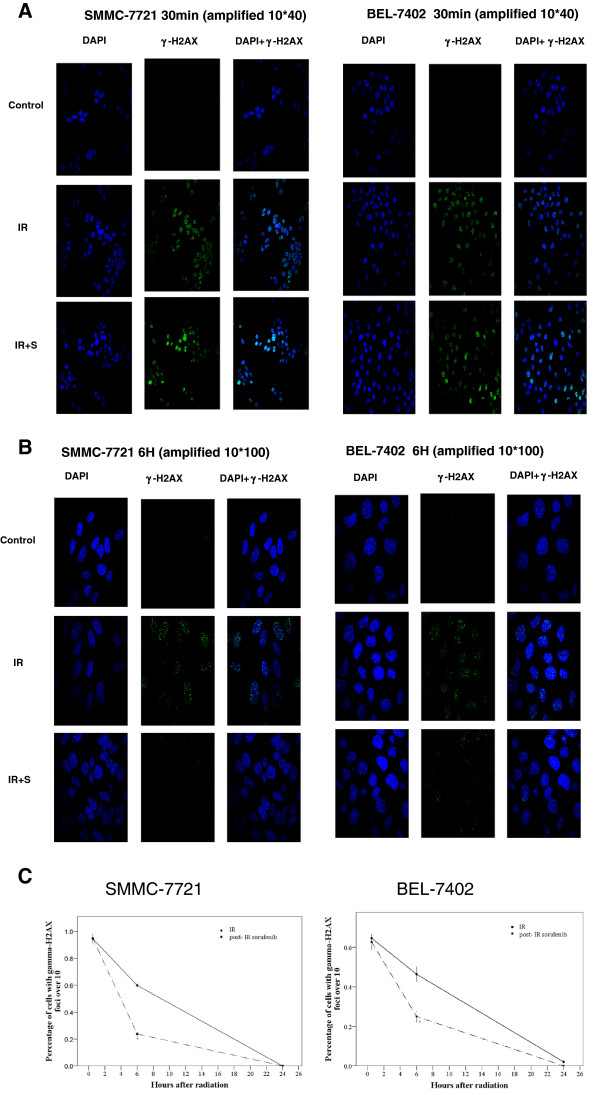
**Effect of sorafenib onDNA damage of irradiatedSMMC-7721 and BEL-7402 cells.** Treated cells were stained with DAPI and anti- γ-H2AX antibody. **A**. Sorafenib was added to SMMC-7721 and BEL-7402 cells 30 min prior to their irradiation (6 Gy). **B**. Post-irradiation sorafenib treated cells were incubated for 6 h before staining. **C**. Percentage of cells with ≥ 10 γ-H2AX foci. Comparisons of mean values were performed using the independent two sample *t* test. Mean ± SD (n = 3). *P < 0.05 vs. the radiation group.

Initially, we hypothesized that pre-radiation sorafenib increased the sensitivity of irradiated hepatocellular carcinoma cells to the formation of DNA double-strand breaks (DSBs). We monitored the formation of DSBs in SMMC-7721 and BEL-7402 cells by examining γ-H2AX induced foci by immunofluorescence. Hepatocellular carcinoma cells were treated with sorafenib for 30 min prior to radiation (6 Gy). Our immunofluorescence assays showed that 94.6 ± 3.5% of irradiated SMMC-7721and 64.7 ± 2.9% of irradiated BEL-7402 cells were positive for γ-H2AX. Similarly, 93.9 ± 4.7% and 62.7 ± 4.0% of SMMC-7721 and BEL-7402 cells that received both radiation and sorafenib were positive for γ-H2AX (Figure 3A to 3C) (*P* > 0.05 in both). These data indicated that pre-irradiation sorafenib did not promote radiation-induced DSBs. We hypothesized that sorafenib may promote the repair of radiation-induced DNA damages. Thus, we compared the percentage of sorafenib-treated (30 min prior), irradiated (6 Gy) cells for γ-H2AX immunofluorescence to radiation treated cells. At 6 h post irradiation, irradiated SMMC-7721 cells had significantly higher γ-H2AX immunofluorescence (59.9 ± 2.4%) than pre-radiation sorafenib-treated, irradiated SMMC-7721 cells (23.8 ± 2.9%) (P < 0.001). Similarly, pre-radiation sorafenib-treated, irradiated BEL-7402 cells had fewer γ-H2AX positive cells (25.0 ± 3.0%) than only irradiated BEL-7402 cells (46.4 ± 3.8%) (P < 0.001) (Figure 3A to 3C).

### Pre-irradiation sorafenib delayed the activation of radiation-induced G2/M checkpoint in hepatocellular carcinoma cells

**Figure 4:**
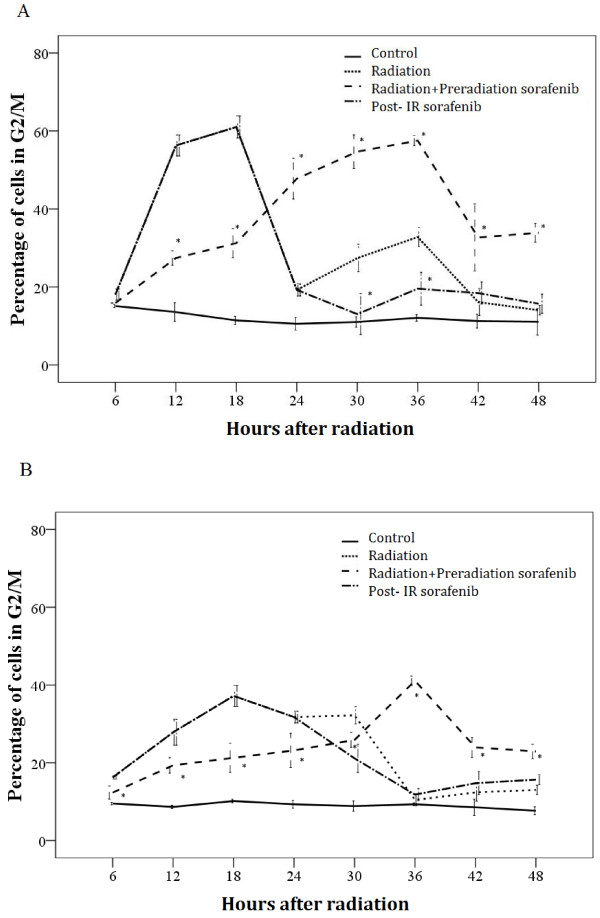
**Effects of sorafenib treatmentson cell cycle distributionof SMMC-7721 and BEL-7402.** Cells were treated with 6 Gy radiation (radiation), 15 μM sorafenib 30 min before 6 Gy radiation (radiation + preradiation sorafenib), or radiation followed 24 hrs later with 15 μM sorafenib (radiation + post radiation sorafenib), or untreated (control). Fixed cells were stained with propidium iodide and analyzed for DNA content by flow cytometry. **A**. SMMC-7721. **B**. BEL-7402. Percentage of hepatocellular carcinoma cells in G2 phase. Comparisons of mean values were performed by using ANOVA. Mean ± SD (n = 3). *P < 0.05 vs. the radiation group.

Radiation-induced DNA damages lead to the activation of G2/M checkpoint. We investigated whether sorafenib given prior to or following irradiation of hepatocellular carcinoma cells impacted radiation-induced changes in distribution of cell cycle stages. Sorafenib alone induced no apparent changes in cell cycle distribution of either SMMC-7721and BEL-7402cells while, as expected, irradiation (6 Gy) caused a significant increase in the percentage of both SMMC-7721 and BEL-7402cells in G2/M at 12 to 16 h post radiation (Figure 4). Pre-irradiation sorafenib also induced an accumulation of the hepatocellular carcinoma cells in G2/M, but this increase in the percentage of cells in G2/M was significantly delayed to 24 to 30 h post irradiation in SMMC-7721 cells and BEL-7402 cells.

### Sorafenib induced apoptosis of hepatocellular carcinoma cells *in vitro*

**Figure 5:**
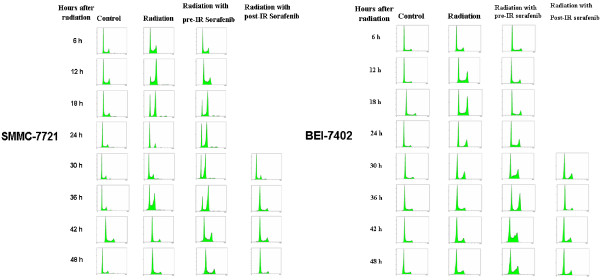
**Effect of sorafenib onapoptosis of irradiated HCCcells.** Sorafenib promoted apoptosis of hepatocellular carcinoma (HCC) cells with or without radiation but had no delaying effect. Cells were treated with 15 μM sorafenib for 30 min prior to 6 Gy irradiation. (**A**) SMMC-7712 cells. (**B**) BEL-7402 cells. Cells were collected at indicated times after the last treatment, and stained with Annexin V-FITC and propidium iodide. Mean ± SEM of three independent experiments.

Sorafenib reduced proliferation of hepatocellular carcinoma cells in CCK8 assays with an IC50 of 25.09 ± 4.49 μM for SMMC-7721 cells and an IC50 of 28.90 ± 1.07 μM for BEL-7402 cells. To examine whether sorafenib induced apoptosis of the hepatocellular carcinoma cells, SMMC-7721and BEL-7402 cells were treated with sorafenib alone. After 24 h, cells were stained with annexin V and propidium iodide to assess percentage of cells undergoing apoptosis. The apoptotic rate in untreated SMMC-7721 (3.4 ± 2.2%) significantly increased more than 4 fold to 18.3 ± 2.9% (P < 0.001) in sorafenib-treated SMMC-7721 (Figure 5A). Sorafenib treatment also increased the apoptotic rate in BEL-7402 cells from 7.2 ± 1.5% to 16.1 ± 2.7% (P < 0.001) (Figure 5B). Radiation did not induce apparent apoptosis of the hepatocellular carcinoma cells SMMC-7721 (6.1 ± 1.0%) compared to controls (4.5 ± 2.3%) or the BEL-7402 cells (8.2 ± 2.1%,vs8.0 ± 1.5% in controls). Interestingly, pre-irradiation sorafenib significantly increased the number of apoptotic cells (SMMC-7721, 18.3 ± 2.0%, *P* < 0.05 vs. controls; BEL-7402, 17.0 ± 2.4%, *P* < 0.05 vs. controls). Post-irradiation sorafenib treatment significantly increased the number of apoptotic cells (SMMC-7721, 15.9 ± 1.8%, *P* < 0.05 vs. controls; BEL-7402, 14.2 ± 2.5%, *P* < 0.05 vs. controls) but to a lesser extent than sorafenib treatment alone. Both pre-irradiation sorafenib and post-irradiation sorafenib induced apoptosis in the hepatocellular cells to a similar extent.

## Discussion

Here, we showed that sorafenib modulated the response of hepatocellular carcinoma cells to radiation and, furthermore, this modulation was schedule-dependent. We found that post-irradiation sorafenib radio sensitized hepatocellular carcinoma cells by inhibiting the clonogenic growth of the hepatocellular carcinoma cells. In contrast, pre-irradiation sorafenib did not radio sensitize these hepatocellular carcinoma cells *in vitro*, which is similar to the findings in colorectal carcinoma [10,11]. Wilson and colleagues [11] investigated the effect of different schedules of sorafenib against irradiated colorectal cancer and pancreatic cancer cells. Only sorafenib given 24 h post irradiation, but not concurrently, potentiated the inhibition of clonogenic growth of irradiated cancer cells [11]. In addition, Plastaras et al. [10] found that radiation alone or sorafenib treatment prior to radiation did not significantly reduce the growth of mouse colorectal cancer xenografts. These above findings suggest that sorafenib exerts a schedule-dependent effect on colorectal carcinoma cells with post-irradiation sorafenib being the most effective in inhibiting tumor growth in mouse models.

Clonogenic cell survival after DNA damage is regulated by two main cell death pathways: interphase apoptotic cell death pathway and mitotic catastrophe [16,18]. Radiation induces mitotic catastrophe [18,19] which occurs in cells with unrepaired DNA damage that prematurely enter mitosis. Mitotic catastrophe is regulated by at least p53, survivin, cell-cycle checkpoint proteins, and cell-cycle specific kinases [20]. To assess whether the schedule-dependent effect of sorafenib on irradiated cells is associated with mitotic catastrophe, we monitored DNA damage in irradiated hepatocellular carcinoma cells by examining γ-H2AX foci with immunofluorescence microscopy. Pre-radiation sorafenib treatment had no effect on the formation of DNA DSBs, but promoted repair of DNA damages, which could lessen the chance of mitotic catastrophe. DNA damage had been almost completely repaired in the irradiated hepatocellular carcinoma cells since less than 5% of the irradiated cells contained significant DNA damage (≥ 10 γ-H2AX foci). We speculate that post-irradiation sorafenib did not increase repair of DNA damages in HCC. The distinct effects on DNA repair by the two schedules of sorafenib may partially explain the enhanced HCC viability with pre-irradiation sorafenib compared to the lower cell viability in irradiated HCC samples treated with sorafenib 24 post radiation.

The activation of cell cycle checkpoints plays a significant role in the DNA damage response. It prevents damaged cells from entering the next phase of the cell cycle. Prolonged G2 arrest appears to contribute to the ability of the cell to survive radiation [21,22]. As expected, we found that irradiation induced the activation of the G2/M checkpoint in hepatocellular carcinoma cells at 16 h post irradiation. Additionally, we observed that pre-irradiation sorafenib delayed the onset of the G2/M checkpoint, which could allow more time for the irradiated hepatocellular carcinoma cells to repair DNA damages. Our clonogenic assays showed that sorafenib given prior to irradiation rendered hepatocellular carcinoma cells more radio resistant, which could be due to the delayed onset of the G2/M checkpoint, allowing the irradiated cells more time to repair DNA damages. As expected, HCC cells treated with post-irradiation sorafenib had no effect on the G2/M peak at 16 hrs post radiation.

As the current study was carried out *in vitro*, we did not examine the anti-angiogenic effect of sorafenib on radio sensitivity in hepatocellular carcinoma cells. We found that sorafenib exerts a schedule-dependent effect on HCC radio sensitivity, which could be of significance for the treatment of hepatocellular carcinoma patients with sorafenib in combination with adjuvant radiotherapy. Our findings suggest that the efficacy of sorafenib-based therapy in combination with radiotherapy may depend on the timing of sorafenib administration relative to that of radiotherapy. On the basis of our *in vitro* studies, we speculate that post-irradiation sorafenib could be more effective in potentiating tumor inhibitory effect of radiotherapy. Further studies are needed to confirm this schedule-dependent effect of sorafenib in animal models bearing human hepatocellular carcinoma xenografts and in clinical studies.

## Conclusions

Sorafenib combined with irradiation exerted a schedule-dependent effect in HCC cells in vitro.Sorafenib given 30 min prior to irradiation reduced the anti-proliferative effects of irradiation against HCC whereas sorafenib given 24 hr after irradiation increased the anti-tumor effects against HCC. These results have significant implications for the combined use of sorafenib and radiotherapy against HCC in the clinic.

## Abbreviations

DSB: Double-strand breaks; HCC: Hepatocellular carcinoma; MTT: 3-(4,5-dimethylthiazol-2-yl)-5(3-carboxymethoxyphenyl)-2(4-sulfophenyl)-2 H-terazolium; OD: Optical density; PBS: Phosphate buffered saline; SD: Standard deviation; SEM: Standard error of the mean.

## Competing interests

None of the authors has any conflict of interest to report.

## Authors' contributions

QL and YH carried out the molecular genetic studies, participated in the sequence alignment and drafted the manuscript. MX carried out the immunoassays. LH participated in the sequence alignment. LZ participated in the design of the study and performed the statistical analysis. ML conceived of the study, participated in its design and coordination, and helped to draft the manuscript. All authors read and approved the final manuscript.

## Pre-publication history

The pre-publication history for this paper can be accessed here:

http://www.biomedcentral.com/1471-2407/12/485/prepub
